# A Runner’s High for New Neurons? Potential Role for Endorphins in Exercise Effects on Adult Neurogenesis

**DOI:** 10.3390/biom11081077

**Published:** 2021-07-21

**Authors:** Timothy J. Schoenfeld, Chance Swanson

**Affiliations:** Department of Psychological Sciences and Neuroscience, Belmont University, Nashville, TN 37212, USA; chance.swanson@pop.belmont.edu

**Keywords:** exercise, adult neurogenesis, beta-endorphin, hippocampus, stress, dentate gyrus, spatial memory, depression

## Abstract

Physical exercise has wide-ranging benefits to cognitive functioning and mental state, effects very closely resembling enhancements to hippocampal functioning. Hippocampal neurogenesis has been implicated in many of these mental benefits of exercise. However, precise mechanisms behind these effects are not well known. Released peripherally during exercise, beta-endorphins are an intriguing candidate for moderating increases in neurogenesis and the related behavioral benefits of exercise. Although historically ignored due to their peripheral release and status as a peptide hormone, this review highlights reasons for further exploring beta-endorphin as a key mediator of hippocampal neurogenesis. This includes possible routes for beta-endorphin signaling into the hippocampus during exercise, direct effects of beta-endorphin on cell proliferation and neurogenesis, and behavioral effects of manipulating endogenous opioid signaling. Together, beta-endorphin appears to be a promising mechanism for understanding the specific ways that exercise promotes adult neurogenesis specifically and brain health broadly.

## 1. Exercise Effects on Brain and Mental Health

Aerobic exercise has widely been prescribed to benefit many physical health conditions, such as cardiovascular disease, obesity, diabetes, and immune functioning [1,2,3], but growing evidence points to chronic aerobic exercise as vital for brain health. Exercise increases blood flow to the brain, reduces risk of stroke, prevents age-associated reductions in brain volume, and is protective against the progression of various neurodegenerative disorders, including Parkinson’s disease, Alzheimer’s disease and other dementias, and progressive multiple sclerosis [4,5,6,7,8,9]. In addition, exercise has wide-ranging positive effects on cognitive functioning and mental health across the lifespan [10,11,12]. Although physical exercise shows strong effectiveness as a prescribed treatment or prevention for many conditions, it is not always practical due to physical and mental barriers behind many neurological and psychiatric conditions [13]. Therefore, having a deeper understanding of the brain mechanisms behind its therapeutic effects is vital to offering the promise of exercise treatment to many.

## 2. Hippocampus and Adult Neurogenesis—Potential Involvement in Many of the Exercise-Related Effects

Many of the cognitive and emotional effects of aerobic exercise involve normalized or enhanced functioning of the hippocampus. Specifically, exercise enhances spatial learning and memory processes, pattern separation and mnemonic discrimination, attention shifting, and negative feedback to stress, in addition to decreasing feelings of anxiety and depression [14,15,16,17,18,19]. Using rodent lesion models, behaviors modeling all of these are dependent on normal hippocampal functioning [20], suggesting that the hippocampus may be a key target to effects of exercise on cognition and mental health. Plastic changes within the hippocampus are likely responsible for producing long-term improvements in brain functioning following chronic exercise, which can be studied in many ways, including dendritic growth of neurons, synapse formation, and long-term potentiation or other changes to physiological strength of synaptic connections. One additional process relatively unique to the hippocampus in the adult mammalian brain is the continual production of new neurons in the dentate gyrus region of the hippocampus. New neurons are born throughout adulthood in the dentate gyrus of mammals, from rodents to primates and humans [21,22], and have the unique potential to dynamically alter hippocampal processing by their introduction to dentate networks.

Recent work utilizing models to ablate neurogenesis in the hippocampus of adult rodents has demonstrated a functional role for adult-born neurons in many of the hippocampal functions that exercise improves. In learning and memory tasks, new neurons in the hippocampus are important for normal adoption of spatial strategies when solving new environments [23] and initial acquisition of contextual fear conditioning [24]. On tasks assessing specific areas of cognitive functioning, deletion of new neurons impairs normal pattern separation abilities [25] and attention shifting [26]. Lastly, with stress physiology and related behaviors, ablating adult neurogenesis impairs negative feedback of the normal corticosterone response to stress and leaves rodents at higher risk for developing anxiety or depression [27], although many studies show that losing new neurons does not simply produce anxiety or depression [28]. Overall, there is much circumstantial evidence to suggest that adult neurogenesis is a possible mechanism behind the benefits of exercise on mental functioning. The purpose of this review is to describe the effects of exercise on adult neurogenesis, discuss the functional relevance of changing adult neurogenesis on exercise’s effects, and highlight β-endorphin as one interesting and underlooked mechanism mediating these effects.

## 3. Exercise Effects on Adult Neurogenesis

Initially, the basic characterization of adult neurogenesis in the hippocampus of rodents occurred in tandem to experiments showing manipulations that reduced cell proliferation and neurogenesis, such as stress. Conversely, living in an enriched environment was shown to enhance adult neurogenesis in mice and follow-up studies demonstrated that access to a running wheel was the most important factor in producing these effects [29]. This important first study and similar ones that followed consistently showed that rodents given free access to running wheels to run whenever they wanted would gain a boost in hippocampal neurogenesis [30,31]. Limiting the timing of wheel access, though, showed that hippocampal neurogenesis would be most promoted when wheel access was granted for multiple hours during the active dark phase of their circadian cycle, when rodents would naturally be most active on running wheels anyway [32].

In general, running longer distances results in increased neurogenesis [33]. However, use of resistance-embedded running wheels results in shorter distances but similar increases to adult neurogenesis [34], suggesting that amount of effort is more importantly associated with neurogenic effects rather than simply distance ran, but to a point. In long-term running conditions (one month or longer), limiting daily access to running wheels results in higher cell proliferation than unlimited access [35,36], suggesting that neurogenesis effects are more nuanced and likely balanced by energy demands exerted by excessive exercise. Relatively newer research looking at different types of exercise on cell proliferation and neurogenesis supports the idea that physical fatigue can offset exercise-induced increases in adult neurogenesis. Adding strength training to a regular treadmill paradigm through body weights and incline walking depresses hippocampal cell proliferation back to baseline levels [37]. However, just performing strength training, as has been conducted with resistance-based ladder climbing, increases cell proliferation on its own [38,39], suggesting that type of exercise—aerobic or anaerobic, endurance or high intensity—may not matter, as long as exercise load does not become excessive. Other resistance-based exercise paradigms show the opposite effect—no change in cell proliferation but decreases in neurogenesis [40], suggesting that disparities in duration and intensity of workouts matter. By contrast, allowing rodents to voluntarily run at their own pace may be the simplest way to keep fatigue in check and maximize hippocampal plasticity.

Voluntary exercise does not universally enhance all facets of adult neurogenesis, however. Transiently, cell proliferation from progenitor cells in the hippocampus is initially suppressed in the first two days of running, although by one week, there are characteristic increases in cell proliferation [41]. New cells are proliferated, but also more new neurons are differentiated and survive to fully integrate into hippocampal circuitry within a matter of weeks [42]. There is even evidence of increased number of new neurons within two days of running [41], likely reflecting enhanced survival or hastened maturation of preexisting neurons [42,43]. However, these effects do not continually persist for the duration of long-term running. By the third week of exercise, running rats no longer have increased cell proliferation [42].

Cell proliferation may initially dip following initiation of running due to stress. Exercise is a physical stressor in that it activates the hypothalamic-pituitary-adrenal (HPA) axis resulting in peripheral increases in corticosterone [44]. Despite consistently inhibitory effects of various stressors, both physical and psychological, on adult neurogenesis (reviewed in [45]), running seems to serve as a ‘positive’ stressor that can maintain beneficial effects on brain plasticity, such as increased adult neurogenesis. One of the ways harmful effects of running stress may be buffered is through social housing. Exercise produces either no change in adult neurogenesis or uncharacteristic decreases in adult neurogenesis in socially isolated rats and mice [46,47,48], and intentionally depressing the level of corticosterone circulating in socially isolated runners restores the beneficial effects of exercise on adult neurogenesis [46]. In addition, the voluntary nature of wheel running is most often utilized as it gives rodents agency in choosing to run and is considered less stressful, compared to prolonged forced exercise on treadmills. Although many studies utilizing treadmills show enhancements in adult neurogenesis [49], intense exercise paradigms using treadmills show diminished increases in neurogenesis [50,51], possibly related to allostatic demands of those paradigms.

## 4. Functional Role of Neurogenesis on Exercise-Induced Changes to Mental Functioning

Although running-increased adult neurogenesis may mediate many of the broad behavioral changes produced by exercise, most studies directly studying the function of new neurons have focused on memory functions of the hippocampus, namely spatial/environmental learning and memory. Overall, effects are mixed when asking whether increased neurogenesis is necessary for improved cognition following exercise. Using irradiation or pharmacogenetic models of neurogenesis inhibition, partial reduction in adult neurogenesis following exercise has been shown to decrease spatial performance in the Morris water maze [52,53]. However, no changes to exercise-enhanced performance were observed in similar studies [54,55,56,57], or following treatment with the anti-mitotic drug, Ara-C [58]. Perhaps surprisingly, one of the papers that found effects of irradiation on exercise-enhanced spatial memory failed to find effects on contextual fear conditioning [52], whereas a paper that failed to find spatial memory deficits did see a reduction in contextual fear conditioning following irradiation [54].

Stress does seem to be an important variable when examining the functional role of new neurons in exercise-induced changes to mental functioning. Although ablation of new neurons does not prevent anxiolysis following exercise in basal conditions [59], loss of new neurons does diminish the reduction in anxiety-like behavior produced by exercise in a chronic pain model [60]. Likewise, even when absence of adult-born neurons in rats fails to impact spatial learning in the Morris water maze following running, it does make running less beneficial when rats were injected with corticosterone [58]. One potential reason why stress may affect hippocampal processing is that stress can saturate hippocampal long-term potentiation (LTP) [61], leading to reduced capacity for future learning. Adult neurogenesis has been proposed to help combat LTP saturation in the hippocampus and maintain hippocampal memory capacity [62]. Important for this discussion, exercise was specifically shown to aid in the recovery of contextual fear learning following artificial LTP saturation, but not when neurogenesis was inhibited by irradiation [62].

It should be noted that many of the cognitive and affective benefits of exercise may be due to other factors outside of changes to cell proliferation and neurogenesis. Long-term running enhances LTP in the dentate gyrus [63] and increases downstream molecular pathways involved in synaptic plasticity [64]. In addition, exercise impacts hippocampal structure in a variety of ways. Exercise increases dendritic length and spine density in hippocampal neurons [65] and increases overall hippocampal volume [66]. Exercise results in the release of many trophic factors, and although there is significant evidence that these growth factors enhance neurogenesis in adult rodents (see below), they are also likely to mediate other physiological and structural changes from exercise [64,67]. Therefore, exercise produces many changes within the hippocampus, and while evidence points to neurogenesis being important for many of the mental benefits of exercise, it is likely one type of plasticity among many that have a functional role.

## 5. Potential Mechanisms Involved in Exercise Effects on Neurogenesis—Endorphins?

Early studies showed various neurotrophic factors important for developmental neurogenesis and neuroprotection mediate exercise effects on adult neurogenesis. Vascular endothelial growth factor (VEGF), released by skeletal muscle cells, is increased by running, promotes cell proliferation in adult rodent neural progenitor pools [68,69] and its activity is necessary for increases in adult neurogenesis [70]. As VEGF promotes angiogenesis within the brain, further studies have shown that only blocking angiogenic activity, through angiotensin II receptor antagonists, is sufficient to block exercise effects on adult neurogenesis [71]. Likewise, insulin-like growth factor I (IGF-I), which is important for cell proliferation and neuronal differentiation and survival in the embryonic brain [72], is necessary for exercise to increase neurogenesis in the adult rodent [73].

The most work, however, has been performed circling the neurotrophic factor BDNF (brain-derived neurotrophic factor). BDNF is expressed relatively weakly during embryonic development, but expression increases postnatally and strongly within the hippocampus [74,75], suggesting it has a role in the process of postnatal neurogenesis. Exercise transiently increases BDNF mRNA expression [76] and BDNF protein increases are consistent across the lifespan within a week [77]. As these levels return to baseline after long-term running, it mirrors effects on cell proliferation that are strongest towards the beginning of running [77]. Through transgenic studies, BDNF is vital for normal neurogenesis in the adult hippocampus [78] and artificially increasing its expression stimulates increases in neurogenesis [79]. In addition, BDNF is required for neurogenesis increases following enriched environment including an exercise wheel [80] and transgenic deletion of the BDNF receptor, TrkB, prevents neurogenesis enhancements by exercise as well [81]. Overall, the consensus suggests BDNF as a key mediator of adult neurogenesis in the adult brain and thus a key mediator of the effects of exercise on neurogenesis as well.

Because running is a physical stressor, it is also tempting to consider what factors may uniquely counteract the well-documented inhibitory effects of negative stressors on adult neurogenesis [45]. One intriguing culprit is the endogenous endorphins, known to be released during aerobic exercise and long thought to be responsible for the proposed ‘runner’s high’ [82], although this specific role may be more suited for exercised-induced release of endocannabinoids [83]. During an acute stressor, hypothalamic corticotropin-releasing hormone (CRH) activates the synthesis of pro-opiomelanocortin (POMC), the precursor for adrenocorticotropic hormone (ACTH) and β-endorphin, both simultaneously released by the anterior pituitary [84]. Traditionally, β-endorphins have a peripheral role in analgesia; helping an organism continue to fight-or-flee despite potential harm and pain from a physical stressor [85]. Intriguingly, though, adult-born neurons have been suggested to be involved in chronic pain states [86] and β-endorphins have also been speculated to be important for a wide array of behaviors and conditions related to hippocampal neurogenesis, including depression, anxiety, and stress physiology [87]. Thus, a more specific role of β-endorphins within the relationship between exercise and adult neurogenesis deserves investigation. Despite many unanswered questions, β-endorphin remains an intriguing candidate for mediating exercise-induced increases to adult neurogenesis [88], as will be outlined here.

## 6. β-Endorphin: Just for the Periphery?

One potential problem with focusing on β-endorphins is the presumed one-way direction of β-endorphin transmission from brain to body. As a peptide hormone, it has long been postulated that β-endorphin is transiently elevated in plasma by stress and exercise via its release from the anterior pituitary but because it does not readily cross the blood–brain barrier, these increases are specific to the periphery. However, the evidence for this largely relies on measurements showing transient drops in β-endorphin levels soon after acute stressors in the hypothalamus and pituitary, from where β-endorphin would be mobilized for secretion to the periphery [89]. It should be noted, however, that β-endorphin is elevated centrally in various brain areas following long-term exercise [90] and mediates behaviors affected by stress [91], suggesting that β-endorphin acts centrally in some capacity during stress and exercise as well. Although it is well known that related opioid peptides, the enkephalins and dynorphins, are synthesized and released locally within the dentate gyrus [92], hippocampal neurons do not functionally express POMC for β-endorphin synthesis [93], suggesting that any β-endorphin signaling would be coming from outside the hippocampus. It is unknown by which mechanisms β-endorphin signaling within the hippocampus occurs, but there at least three possible routes: (1) β-endorphin may be released centrally by axons projecting from POMC-expressing hypothalamic neurons, (2) β-endorphin may be transported across the blood–brain barrier after being released peripherally, or (3) β-endorphin may be secreted into cerebrospinal fluid (CSF) and transported to the hippocampus via volume transmission in cerebral ventricles.

The most direct route of β-endorphin activity within the dentate gyrus would be from direct innervations of β-endorphin-releasing axon terminals in the dentate gyrus. Neurons that use β-endorphin are typically labeled through their expression of POMC and using autoradiographic and immunofluorescent methods, POMC neurons have been primarily localized within the hypothalamus, specifically the arcuate nucleus [94,95]. Viral tracing studies conclude that POMC neurons in the hypothalamus do not directly innervate the dentate gyrus [95], showing that direct transmission of β-endorphin from hypothalamic POMC neurons is unlikely. Blocking β-endorphin activity in the brain through antibody treatment does alter glucose metabolism in the dentate gyrus during active pain [96], showing that β-endorphin has a modulatory role in dentate gyrus activity patterns. Although, if this is due to central release of β-endorphin from POMC neurons, it is likely indirect through activity of extrahippocampal areas, such as the amygdala.

β-endorphin protein expression is still reported within the dentate gyrus and its expression can be modified by experience [97], so more indirect routes of β-endorphin signaling are likely. One possibility for this is transport of β-endorphin from plasma across the blood–brain barrier into the dentate gyrus specifically or CSF broadly. Experiments injecting radioactive β-endorphin intravenously into rats show a steady increase in intact radioactive β-endorphin in CSF over 90 min [98], suggesting that this mechanism is possible, albeit slow. A few mechanisms may help β-endorphin cross the blood–brain barrier where it is not naturally soluble. The organic anion-transporting polypeptide (Oatp) family of membrane transporters have been shown to transport opioid peptides across the blood–brain barrier [99], although whether β-endorphin is one of them or if transporter activity is regulated by exercise is still unclear. In addition, β-endorphin has been demonstrated to be transportable through bonding with other peptides or molecules that are transportable [100]. However, evidence of this occurring naturally is still lacking. It seems feasible that plasma β-endorphin can cross back into the central nervous system. Although this route may be too slow to reliably affect behaviors following acute stress or exercise, it may be well suited for neuroprotection of cell proliferation and neurogenesis during chronic running conditions.

**Figure 1:**
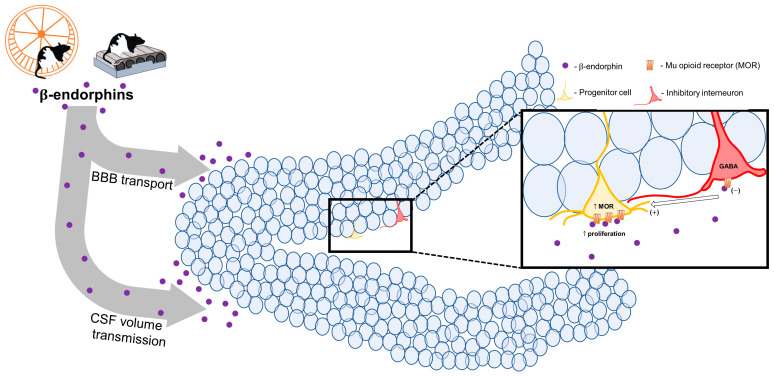
Proposed β-endorphin influence on adult neurogenesis following exercise. During multiple types of exercise, β-endorphins are released peripherally, which may signal in the dentate gyrus either through transport across the blood–brain barrier (BBB) or direct infusion into cerebrospinal fluid (CSF) and to the hippocampus via volume transmission. Once at the dentate gyrus, β-endorphins may enhance cell proliferation by both directly inducing proliferative activity in progenitor cells from acting on mu opioid receptors (MOR), which upregulate during exercise, or enhancing local net excitation by inhibiting GABAergic interneurons.

One last route β-endorphin can take is by skipping the periphery and going into CSF directly. Peptides secreted by arcuate nucleus neurons may directly feed into CSF [101], and indeed β-endorphin is elevated in CSF following exercise [102]. Plasma and CSF concentrations of β-endorphin do not increase and decrease in parallel, either under baseline or following stress or exercise, suggesting that the two routes of β-endorphin release are independent of one another [103]. Once secreted into CSF, β-endorphin needs to exit the ventricles and be transported into hippocampal tissue, and there is evidence that ventricular signals can do just that [104]. Additional evidence that intracerebroventricular injections of β-endorphin induce the greatest increase in glucose usage in the hippocampus [105] suggests that β-endorphin may act directly within the hippocampus following transport into CSF. Thus, in addition to possible blood–brain barrier transport, β-endorphin may be released directly into CSF centrally to influence expression in the hippocampus. Much of this remains to be directly tested. However, elevated β-endorphin from either of these two routes has the possibility to act within the hippocampus and moderate cell proliferation and/or neurogenesis and neuron survival once there (Figure 1).

## 7. Direct Evidence for Endorphin Influence on Neurogenesis

The most direct evidence for β-endorphin in mediating exercise-induced increases in adult neurogenesis comes from β-endorphin knockout mice. Mice without β-endorphin were prevented from increases in cell proliferation in the hippocampus from wheel running [106]. These effects on cell proliferation held for both short-term (10 days) and long-term (39 days) running, although surprisingly, neurogenesis and cell survival remained enhanced in runners without β-endorphin for both durations [106], suggesting that other mechanisms can still promote neurogenesis with longer bouts of exercise, likely through enhancing cell survival [42]. Importantly, β-endorphin-deficient mice maintained normal degrees of cell proliferation and neurogenesis in sedentary conditions, showing that β-endorphin is uniquely involved in exercise-effects on adult neurogenesis [106].

β-endorphin acts on multiple opioid receptors in the brain, namely the mu-opioid receptor (MOR) and delta-opioid receptor (DOR). Peripheral administration of naltrexone, a MOR-preferential antagonist, increases cell proliferation in the hippocampus of sedentary rats, but suppresses the enhanced cell proliferation of running rats [107]. For sedentary rats, these effects are likely due to inhibition of HPA-axis activation [107,108]. However, for running rats, these effects may reflect β-endorphin acting on MORs as a mediator of exercise-produced neurogenesis. In cultured adult hippocampal progenitors, MORs exist in their cell membrane and their activation induces proliferative pathways [109], suggesting that MOR activity by β-endorphins would stimulate cell proliferation in a vacuum, through activation of mitogen-activated protein kinase (MAPK) pathways [110]. In addition, β-endorphin increases BDNF mRNA expression in the dentate gyrus, effects that are blocked by naltrexone as well [111] and show that β-endorphins can enhance cell proliferation and adult neurogenesis through indirect means too. These effects seem to be specific to the MOR, as drugs that are antagonists of DOR do not show the same effects. Interestingly, exercise enhances MOR expression within the dentate gyrus quickly (within 5 days of running) [112], so even if β-endorphin levels return to baseline during chronic running, changes to MOR expression may continue to moderate proliferative effects.

Instead of acting directly on progenitor cells, β-endorphin may act to promote cell proliferation indirectly through GABAergic interneurons in the dentate gyrus. Broadly, endogenous opioid peptides increase activation of the dentate gyrus [113], largely through inhibiting local GABAergic interneurons to increase net excitation [114,115]. Because progenitor cells and new neuroblasts in the dentate gyrus contain GABA receptors, proliferation can be enhanced through disinhibition of GABA [116]. These effects may be short lived, however. Long-term running enhances GABAergic signaling within the dentate gyrus [31], which likely aids in long-term survival and growth of new neurons [117], so the inhibitory effects of β-endorphin on GABAergic interneurons are compensated for at some point. This may be due to a corrective return of MORs to baseline levels in long-term running conditions [112] or habituation in running-induced release of β-endorphin peripherally or centrally. However, this has not been directly investigated.

## 8. Circumstantial Evidence for β-Endorphin Roles in Neurogenesis—A Need for More Behavioral Studies Utilizing Endogenous Opioids

If β-endorphin release during exercise directly promotes neurogenesis increases in adult rodents, β-endorphin should cause behavioral changes indicative of increasing neurogenesis in the hippocampus of adult rodents alone. This is problematic for two reasons. First, behavioral changes have been somewhat difficult to find by solely increasing neurogenesis, independent of experiences such as environmental enrichment or exercise. For example, utilizing a transgenic mouse with induced deletion of a *Bax* gene that promotes apoptosis allows for artificially enhanced neurogenesis and cell survival in the dentate gyrus. With this model, mice have shown enhanced pattern separation under baseline conditions and reduced stress physiology and depressive-like behaviors only under chronic stress conditions [118,119,120]. Although these experiments show functional benefits of increasing neurogenesis in the hippocampus, there has not yet been the wide range of functional effects seen through ablation studies or through enhanced neurogenesis by experiences such as exercise, as described earlier.

Second, the picture becomes even more difficult when trying to directly relate these behavioral effects of artificially enhanced neurogenesis to the increases in neurogenesis through β-endorphin signaling that would occur during exercise. The majority of studies examining the effects of β-endorphin on behaviors related to hippocampal neurogenesis, such as spatial learning and memory and behaviors related to stress and mood use acute models to study real-time effects of injected β-endorphin into experimental rodents [111,121,122]. This is difficult not just because rodent exercise effects involve chronically elevated β-endorphin levels for weeks, but because any behavioral effects dependent on enhanced neurogenesis would also likely require weeks for new neurons to integrate into dentate gyrus circuitry, similar to effects of antidepressants [123]. However, chronic β-endorphin treatment studies are currently lacking.

If β-endorphin studies like this are lacking, it could be informative to look at other opioid treatments that act on the same receptors that β-endorphin does. Problematically, chronic exogenous opioid administration through morphine injections largely suppresses hippocampal cell proliferation and neurogenesis [124] and induces a depressive state [125] suggesting negative effects in absence of physical exercise. However, chronic treatment with dermorphin, a MOR-specific agonist, decreases elevations of corticosterone following stress [126]. Similar to β-endorphin effects on BDNF, these effects seem to be only for the MOR, as DOR agonists do not affect corticosterone levels [126], and in all suggests that the suppressive effects of chronic morphine administration can be dissociable from effects of β-endorphin, perhaps through differences in opioid receptor activity. Additionally, experiments using opioid receptor antagonists throughout exercise would be helpful. However, these experiments are lacking. Although naloxone, a non-specific opioid receptor antagonist, exacerbates increased cell proliferation during treadmill running, which could imply that β-endorphin signaling during exercise is actually suppressive on neurogenesis [127], these effects are only after acute bouts of running, making it difficult to compare directly to chronic running and β-endorphin effects.

It would be tempting to consider β-endorphin knockout rodents as evidence towards a link between adult neurogenesis and β-endorphin signaling, particularly when β-endorphin knockout mice show similar behavioral traits as neurogenesis-inhibited mice [128,129]. However, because β-endorphin knockouts have similar neurogenesis rates to wildtype controls [106], these models would be most helpful under exercise conditions, when neurogenesis would be affected by reduced β-endorphin. Unfortunately, experiments investigating β-endorphin knockouts under exercise conditions have only explored analgesic and addictive properties of β-endorphin [85,130], behaviors not primarily implicating hippocampal neurogenesis, so there is a lack of direct evidence in this domain. The opposite models, enhanced β-endorphin, would be helpful to show positive effects of running without the confounding additional physiological effects of exercise. Interestingly, an adenovirus has recently been created which chronically enhances β-endorphin levels centrally [131]. However, it is too soon to know whether this model can be used to imitate effects of running on behaviors related to the hippocampus and adult neurogenesis.

## 9. Implications and Conclusions

To better establish the connection between exercise-induced increases in β-endorphin and adult neurogenesis in the dentate gyrus, further studies are recommended. First, the mechanisms by which β-endorphin enter and act in the hippocampus following periods of exercise need to be established. Concurrently, how the actions of β-endorphin in the dentate gyrus potentially change longitudinally during long-term running, in both the levels of β-endorphin present and pharmacodynamics of it, would be important to connect to what is known about changes in adult neurogenesis over the same time frame. Second, chronic studies utilizing long-term increases in β-endorphin, peripherally or centrally, should be performed to see if solely increasing β-endorphin can mimic exercise effects on either hippocampal behaviors or adult neurogenesis. Third, long-term studies combining chronic exercise and either opioid receptor antagonists or β-endorphin knockouts should be used to see if blocking endogenous opioid signaling during exercise affects either hippocampal behaviors or adult neurogenesis.

Finally, growing research suggests that not only are there sex effects on exercise, endorphins, and the hippocampus, but that sex steroid hormones themselves mediate the effects of opioid peptides. Evidence in humans suggest that men and women have different β-endorphin levels peripherally during certain periods of aerobic exercise [132]. These potential sex differences in β-endorphin release may explain some sex differences in exercise effects on behavior. For example, male and female rodents show different degrees of cross-tolerance to morphine following chronic exercise [133], stress physiology and depressive behavior [134,135,136,137], and spatial and non-spatial learning and memory [138]. Despite behavioral differences, hippocampal cell proliferation is similarly increased by exercise in male and female rats [46,47], although there have been sex differences observed in neurogenesis and survival of new neurons generated during exercise [139,140]. How sex effects on β-endorphin release during exercise might functionally impact hippocampal neurogenesis and/or behavior is unknown, but these possibilities deserve direct investigation moving forward. Across both sexes, sex steroid hormones modulate central β-endorphin, including the hippocampus [141,142], suggesting that there can be a wide variability of β-endorphin even within the sexes based on timing and experience. How these hormone effects influence exercise-induced concentrations of β-endorphin is currently unknown, save for differences between female athletes based on ovulatory status [143], and deserve further exploration.

From studies that have been conducted, it seems that under baseline conditions, β-endorphin and endogenous opioid signaling affect adult neurogenesis (and by proxy hippocampal physiology and behavior) minimally. However, under stressful conditions, the actions of endogenous opioids are more pronounced. Naloxone administration worsens the effects of acute stress on learning [144], suggesting that endogenous opioids may help buffer the aversive effects of stress. Additionally, although blocking endogenous opioid activity during stress with naltrexone fails to prevent stress-induced decreases in hippocampal cell proliferation [145], the opposite may very well be true—endogenous opioid activity may uniquely buffer the dentate gyrus from the effects of running stress on cell proliferation to allow for greater neurogenesis. This preservation and even enhancement of adult neurogenesis despite the physical stress of running may provide ethological advantages to rodents in the wild, similar to how endogenous opioids help to keep muscle pain low during extended runs [146]. Stress-filled environments generally produce reductions to adult neurogenesis and bias rodent behavior towards more caution—lower degrees of exploration and foraging and higher degrees of novelty avoidance—all of which may be beneficial for survival amid an unpredictable or dangerous environment [147]. By contrast, rodents with high levels of physical activity are likely freely exploring their environments, so diminished exploration and spatial learning through lower neurogenesis would be maladaptive. Therefore, having a mechanism through β-endorphin to mitigate the negative effects of stress on adult neurogenesis during running may continue to foster a hippocampal environment that biases reward seeking and confidence in a richer and safer environment [148]. For humans, the potentially positive effects of β-endorphin signaling on hippocampal plasticity further highlights the importance of exercise as an everyday experience and treatment option for a variety of physical and mental conditions. Further, increased experimentation detailing how β-endorphin is functionally involved in promoting hippocampal plasticity provides avenues for the development of new treatments for those for whom regular exercise is difficult or impossible to partake.

## References

[1] Jesus I., Vanhee V., Deramaudt T.B., Bonay M. (2021). Promising effects of exercise on the cardiovascular, metabolic and immune system during COVID-19 period. J. Hum. Hypertens..

[2] Gorostegi-Anduaga I., Corres P., MartinezAguirre-Betolaza A., Pérez-Asenjo J., Aispuru G.R., Fryer S.M., Maldonado-Martín S. (2018). Effects of different aerobic exercise programmes with nutritional intervention in sedentary adults with overweight/obesity and hypertension: EXERDIET-HTA study. Eur. J. Prev. Cardiol..

[3] Al Dahamsheh Z., Al Rashdan K., Al Hadid A., Jaradat R., Al Bakheet M., Bataineh Z.S. (2019). The Impact of Aerobic Exercise on Female Bone Health Indicators. Med. Arch..

[4] Howard V.J., McDonnell M.N. (2015). Physical Activity in Primary Stroke Prevention: Just Do It!. Stroke.

[5] Ainslie P.N., Cotter J.D., George K.P., Lucas S., Murrell C., Shave R., Thomas K.N., Williams M.J.A., Atkinson G. (2008). Elevation in cerebral blood flow velocity with aerobic fitness throughout healthy human ageing. J. Physiol..

[6] Colcombe S.J., Erickson K.I., Scalf P.E., Kim J.S., Prakash R., McAuley E., Elavsky S., Marquez D.X., Hu L., Kramer A.F. (2006). Aerobic exercise training increases brain volume in aging humans. J. Gerontol. Ser. A Biol. Sci. Med. Sci..

[7] Ahlskog J.E. (2011). Does vigorous exercise have a neuroprotective effect in Parkinson disease?. Neurology.

[8] Paillard T., Rolland Y., de Souto Barreto P. (2015). Protective Effects of Physical Exercise in Alzheimer’s Disease. J. Clin. Neurol..

[9] Briken S., Gold S.M., Patra S., Vettorazzi E., Harbs D., Tallner A., Ketels G., Schulz K.H., Heesen C. (2014). Effects of exercise on fitness and cognition in progressive MS: A randomized, controlled pilot trial. Mult. Scler. J..

[10] Hillman C.H., Erickson K.I., Kramer A.F. (2008). Be smart, exercise your heart: Exercise effects on brain and cognition. Nat. Rev. Neurosci..

[11] Dilorenzo T.M., Bargman E.P., Stucky-Ropp R., Brassington G.S., Frensch P.A., LaFontaine T. (1999). Long-term effects of aerobic exercise on psychological outcomes. Prev. Med..

[12] Kandola A., Hendrikse J., Lucassen P.J., Yücel M. (2016). Aerobic Exercise as a Tool to Improve Hippocampal Plasticity and Function in Humans: Practical Implications for Mental Health Treatment. Front. Hum. Neurosci..

[13] Way K., Kannis-Dymand L., Lastella M., Lovell G.P. (2018). Mental health practitioners’ reported barriers to prescription of exercise for mental health consumers. Ment. Health Phys. Act..

[14] Bernstein E.E., McNally R.J. (2019). Examining the Effects of Exercise on Pattern Separation and the Moderating Effects of Mood Symptoms. Behav. Ther..

[15] Erickson K.I., Voss M.W., Prakash R.S., Basak C., Szabo A., Chaddock L., Kim J.S., Heo S., Alves H., White S.M. (2011). Exercise training increases size of hippocampus and improves memory. Proc. Natl. Acad. Sci. USA.

[16] Petruzzello S.J., Landers D.M., Hatfield B.D., Kubitz K.A., Salazar W. (1991). A Meta-Analysis on the Anxiety-Reducing Effects of Acute and Chronic Exercise: Outcomes and Mechanisms. Sports Med..

[17] Craft L.L., Landers D.M. (1998). The Effect of Exercise on Clinical Depression and Depression Resulting from Mental Illness: A Meta-Analysis. J. Sport Exerc. Psychol..

[18] Zschucke E., Renneberg B., Dimeo F., Wüstenberg T., Ströhle A. (2015). The stress-buffering effect of acute exercise: Evidence for HPA axis negative feedback. Psychoneuroendocrinology.

[19] Masley S., Roetzheim R., Gualtieri T. (2009). Aerobic exercise enhances cognitive flexibility. J. Clin. Psychol. Med. Settings.

[20] Cameron H.A., Glover L.R. (2015). Adult Neurogenesis: Beyond Learning and Memory. Annu. Rev. Psychol..

[21] Gould E. (2007). How widespread is adult neurogenesis in mammals?. Nat. Rev. Neurosci..

[22] Moreno-Jimenez E.P., Terreros-Roncal J., Flor-Garcia M., Rabano A., Llorens-Martin M. (2021). Evidences for Adult Hippocampal Neurogenesis in Humans. J. Neurosci..

[23] Garthe A., Kempermann G. (2013). An old test for new neurons: Refining the morris water maze to study the functional relevance of adult hippocampal neurogenesis. Front. Neurosci..

[24] Drew M.R., Denny C.A., Hen R. (2010). Arrest of adult hippocampal neurogenesis in mice impairs single- but not multiple-trial contextual fear conditioning. Behav. Neurosci..

[25] Clelland C.D., Choi M., Romberg C., Clemenson G.D., Fragniere A., Tyers P., Jessberger S., Saksida L.M., Barker R.A., Gage F.H. (2009). A functional role for adult hippocampal neurogenesis in spatial pattern separation. Science.

[26] Weeden C.S.S., Mercurio J.C., Cameron H.A. (2019). A role for hippocampal adult neurogenesis in shifting attention toward novel stimuli. Behav. Brain Res..

[27] Snyder J.S., Soumier A., Brewer M., Pickel J., Cameron H.A. (2011). Adult hippocampal neurogenesis buffers stress responses and depressive behaviour. Nature.

[28] Schoenfeld T.J., Cameron H.A. (2015). Adult neurogenesis and mental illness. Neuropsychopharmacology.

[29] Van Praag H., Kempermann G., Gage F.H. (1999). Running increases cell proliferation and neurogenesis in the adult mouse dentate gyrus. Nat. Neurosci..

[30] Brown J., Cooper-Kuhn C.M., Kempermann G., Van Praag H., Winkler J., Gage F.H., Kuhn H.G. (2003). Enriched environment and physical activity stimulate hippocampal but not olfactory bulb neurogenesis. Eur. J. Neurosci..

[31] Schoenfeld T.J., Rada P., Pieruzzini P.R., Hsueh B., Gould E. (2013). Physical exercise prevents stress-induced activation of granule neurons and enhances local inhibitory mechanisms in the dentate gyrus. J. Neurosci..

[32] Holmes M.M., Galea L.A.M., Mistlberger R.E., Kempermann G. (2004). Adult Hippocampal Neurogenesis and Voluntary Running Activity: Circadian and Dose-Dependent Effects. J. Neurosci. Res..

[33] Rhodes J.S., Jeffrey S., Girard I., Mitchell G.S., Van Praag H., Garland T., Gage F.H. (2003). Exercise increases hippocampal neurogenesis to high levels but does not improve spatial learning in mice bred for increased voluntary wheel running. Behav. Neurosci..

[34] Lee M.C., Inoue K., Okamoto M., Liu Y.F., Matsui T., Yook J.S., Soya H. (2013). Voluntary resistance running induces increased hippocampal neurogenesis in rats comparable to load-free running. Neurosci. Lett..

[35] Nguemeni C., McDonald M.W., Jeffers M.S., Livingston-Thomas J., Lagace D., Corbett D. (2018). Short- and Long-term Exposure to Low and High Dose Running Produce Differential Effects on Hippocampal Neurogenesis. Neuroscience.

[36] Huang Y.Q., Wu C., He X.F., Wu D., He X., Liang F.Y., Dai G.Y., Pei Z., Xu G.Q., Lan Y. (2018). Effects of voluntary wheel-running types on hippocampal neurogenesis and spatial cognition in middle-aged mice. Front. Cell. Neurosci..

[37] Lan Y., Huang Z., Jiang Y., Zhou X., Zhang J., Zhang D., Wang B., Hou G. (2018). Strength exercise weakens aerobic exerciseinduced cognitive improvements in rats. PLoS ONE.

[38] Codina-Martínez H., Fernández-García B., Díez-Planelles C., Fernández Á.F., Higarza S.G., Fernández-Sanjurjo M., Díez-Robles S., Iglesias-Gutiérrez E., Tomás-Zapico C. (2020). Autophagy is required for performance adaptive response to resistance training and exercise-induced adult neurogenesis. Scand. J. Med. Sci. Sports.

[39] Novaes Gomes F.G., Fernandes J., Vannucci Campos D., Cassilhas R.C., Viana G.M., D’Almeida V., de Moraes Rêgo M.K., Buainain P.I., Cavalheiro E.A., Arida R.M. (2014). The beneficial effects of strength exercise on hippocampal cell proliferation and apoptotic signaling is impaired by anabolic androgenic steroids. Psychoneuroendocrinology.

[40] Nokia M.S., Lensu S., Ahtiainen J.P., Johansson P.P., Koch L.G., Britton S.L., Kainulainen H. (2016). Physical exercise increases adult hippocampal neurogenesis in male rats provided it is aerobic and sustained. J. Physiol..

[41] Gremmelspacher T., Gerlach J., Hubbe A., Haas C.A., Häussler U. (2017). Neurogenic processes are induced by very short periods of voluntary wheel-running in male mice. Front. Neurosci..

[42] Snyder J.S., Glover L.R., Sanzone K.M., Kamhi J.F., Cameron H.A. (2009). The effects of exercise and stress on the survival and maturation of adult-generated granule cells. Hippocampus.

[43] Sah N., Peterson B.D., Lubejko S.T., Vivar C., Van Praag H. (2017). Running reorganizes the circuitry of one-week-old adult-born hippocampal neurons. Sci. Rep..

[44] Stranahan A.M., Lee K., Mattson M.P. (2008). Central mechanisms of HPA axis regulation by voluntary exercise. Neuromolecular Med..

[45] Schoenfeld T.J., Gould E. (2012). Stress, stress hormones, and adult neurogenesis. Exp. Neurol..

[46] Stranahan A.M., Khalil D., Gould E. (2006). Social isolation delays the positive effects of running on adult neurogenesis. Nat. Neurosci..

[47] Leasure J.L., Decker L. (2009). Social isolation prevents exercise-induced proliferation of hippocampal progenitor cells in female rats. Hippocampus.

[48] Hauser T., Klaus F., Lipp H.P., Amrein I. (2009). No effect of running and laboratory housing on adult hippocampal neurogenesis in wild caught long-tailed wood mouse. BMC Neurosci..

[49] Li H., Liang A., Guan F., Fan R., Chi L., Yang B. (2013). Regular treadmill running improves spatial learning and memory performance in young mice through increased hippocampal neurogenesis and decreased stress. Brain Res..

[50] Inoue K., Okamoto M., Shibato J., Lee M.C., Matsui T., Rakwal R., Soya H. (2015). Long-term mild, rather than intense, exercise enhances adult hippocampal neurogenesis and greatly changes the transcriptomic profile of the hippocampus. PLoS ONE.

[51] Okamoto M., Yamamura Y., Liu Y.-F., Min-Chul L., Matsui T., Shima T., Soya M., Takahashi K., Soya S., McEwen B.S. (2016). Hormetic effects by exercise on hippocampal neurogenesis with glucocorticoid signaling. Brain Plast..

[52] Clark P.J., Brzezinska W.J., Thomas M.W., Ryzhenko N.A., Toshkov S.A., Rhodes J.S. (2008). Intact neurogenesis is required for benefits of exercise on spatial memory but not motor performance or contextual fear conditioning in C57BL/6J mice. Neuroscience.

[53] Winocur G., Becker S., Luu P., Rosenzweig S., Wojtowicz J.M. (2012). Adult hippocampal neurogenesis and memory interference. Behav. Brain Res..

[54] Wojtowicz J.M., Askew M.L., Winocur G. (2008). The effects of running and of inhibiting adult neurogenesis on learning and memory in rats. Eur. J. Neurosci..

[55] Wong-Goodrich S.J.E., Pfau M.L., Flores C.T., Fraser J.A., Williams C.L., Jones L.W. (2010). Voluntary running prevents progressive memory decline and increases adult hippocampal neurogenesis and growth factor expression after whole-brain irradiation. Cancer Res..

[56] Hamilton G.F., Majdak P., Miller D.S., Bucko P.J., Merritt J.R., Krebs C.P., Rhodes J.S. (2016). Evaluation of a C57BL/6J x 129S1/SvImJ Hybrid Nestin-Thymidine Kinase Transgenic Mouse Model for Studying the Functional Significance of Exercise-Induced Adult Hippocampal Neurogenesis. Brain Plast..

[57] Snyder J.S., Cahill S.P., Frankland P.W. (2017). Running Promotes Spatial Bias Independently of Adult Neurogenesis. Hippocampus.

[58] Yau S.Y., Lau B.W.M., Bin Tong J., Wong R., Ching Y.P., Qiu G., Tang S.W., Lee T.M.C., So K.F. (2011). Hippocampal neurogenesis and dendritic plasticity support running-improved spatial learning and depression-like behaviour in stressed rats. PLoS ONE.

[59] Schoenfeld T.J., McCausland H.C., Sonti A.N., Cameron H.A. (2016). Anxiolytic Actions of Exercise in Absence of New Neurons. Hippocampus.

[60] Zheng J., Jiang Y.Y., Xu L.C., Ma L.Y., Liu F.Y., Cui S., Cai J., Liao F.F., Wan Y., Yi M. (2017). Adult hippocampal neurogenesis along the dorsoventral axis contributes differentially to environmental enrichment combined with voluntary exercise in alleviating chronic inflammatory pain in mice. J. Neurosci..

[61] Kim J.J., Foy M.R., Thompson R.F. (1996). Behavioral stress modifies hippocampal plasticity through N-methyl-D-asparate receptor activation. Proc. Natl. Acad. Sci. USA.

[62] Alam M.J., Kitamura T., Saitoh Y., Ohkawa N., Kondo T., Inokuchi K. (2018). Adult neurogenesis conserves hippocampal memory capacity. J. Neurosci..

[63] Patten A.R., Sickmann H., Hryciw B.N., Kucharsky T., Parton R., Kernick A., Christie B.R. (2013). Long-term exercise is needed to enhance synaptic plasticity in the hippocampus. Learn. Mem..

[64] Vaynman S., Ying Z., Gomez-Pinilla F. (2004). Hippocampal BDNF mediates the efficacy of exercise on synaptic plasticity and cognition. Eur. J. Neurosci..

[65] Eadie B.D., Redila V.A., Christie B.R. (2005). Voluntary exercise alters the cytoarchitecture of the adult dentate gyrus by increasing cellular proliferation, dendritic complexity, and spine density. J. Comp. Neurol..

[66] Biedermann S., Fuss J., Zheng L., Sartorius A., Falfán-Melgoza C., Demirakca T., Gass P., Ende G., Weber-Fahr W. (2012). In vivo voxel based morphometry: Detection of increased hippocampal volume and decreased glutamate levels in exercising mice. NeuroImage.

[67] Wang R., Holsinger R.M.D. (2018). Exercise-induced brain-derived neurotrophic factor expression: Therapeutic implications for Alzheimer’s dementia. Ageing Res. Rev..

[68] Jin K., Zhu Y., Sun Y., Mao X.O., Xie L., Greenberg D.A. (2002). Vascular endothelial growth factor (VEGF) stimulates neurogenesis in vitro and in vivo. Proc. Natl. Acad. Sci. USA.

[69] Rich B., Scadeng M., Yamaguchi M., Wagner P.D., Breen E.C. (2017). Skeletal myofiber vascular endothelial growth factor is required for the exercise training-induced increase in dentate gyrus neuronal precursor cells. J. Physiol..

[70] Fabel K., Fabel K., Tam B., Kaufer D., Baiker A., Simmons N., Kuo C.J., Palmer T.D. (2003). VEGF is necessary for exercise-induced adult hippocampal neurogenesis. Eur. J. Neurosci..

[71] Mukuda T., Sugiyama H. (2007). An angiotensin II receptor antagonist suppresses running-enhanced hippocampal neurogenesis in rat. Neurosci. Res..

[72] Nieto-Estévez V., Defterali Ç., Vicario-Abejón C. (2016). IGF-I: A key growth factor that regulates neurogenesis and synaptogenesis from embryonic to adult stages of the brain. Front. Neurosci..

[73] Trejo J.L., LLorens-Martín M.V., Torres-Alemán I. (2008). The effects of exercise on spatial learning and anxiety-like behavior are mediated by an IGF-I-dependent mechanism related to hippocampal neurogenesis. Mol. Cell. Neurosci..

[74] Hofer M., Hofer M., Pagliusi S.R., Pagliusi S.R., Hohn A., Hohn A., Leibrock J., Leibrock J., Barde Y., Barde Y. (1990). Regional distribution. EMBO J..

[75] Bath K.G., Akins M.R., Lee F.S. (2012). BDNF control of adult SVZ neurogenesis. Dev. Psychobiol..

[76] Oliff H.S., Berchtold N.C., Isackson P., Cotman C.W. (1998). Exercise-induced regulation of brain-derived neurotrophic factor (BDNF) transcripts in the rat hippocampus. Mol. Brain Res..

[77] Adlard P.A., Perreau V.M., Cotman C.W. (2005). The exercise-induced expression of BDNF within the hippocampus varies across life-span. Neurobiol. Aging.

[78] Lee J., Duan W., Mattson M.P. (2002). Evidence that brain-derived neurotrophic factor is required for basal neurogenesis and mediates, in part, the enhancement of neurogenesis by dietary restriction in the hippocampus of adult mice. J. Neurochem..

[79] Quesseveur G., David D.J., Gaillard M.C., Pla P., Wu M.V., Nguyen H.T., Nicolas V., Auregan G., David I., Dranovsky A. (2013). BDNF overexpression in mouse hippocampal astrocytes promotes local neurogenesis and elicits anxiolytic-like activities. Transl. Psychiatry.

[80] Rossi C., Angelucci A., Costantin L., Braschi C., Mazzantini M., Babbini F., Fabbri M.E., Tessarollo L., Maffei L., Berardi N. (2006). Brain-derived neurotrophic factor (BDNF) is required for the enhancement of hippocampal neurogenesis following environmental enrichment. Eur. J. Neurosci..

[81] Li Y., Luikart B.W., Birnbaum S., Chen J., Kwon C.H., Kernie S.G., Bassel-Duby R., Parada L.F. (2008). TrkB Regulates Hippocampal Neurogenesis and Governs Sensitivity to Antidepressive Treatment. Neuron.

[82] Wildmann J., Kruger A., Schmole M., Niemann J., Matthaei H. (1986). Increase of circulating beta-endorphin-like immunoreactivity correlates with the change in feeling of pleasantness after running. Life Sci..

[83] Fuss J., Steinle J., Bindila L., Auer M.K., Kirchherr H., Lutz B., Gass P. (2015). A runner’s high depends on cannabinoid receptors in mice. Proc. Natl. Acad. Sci. USA.

[84] Guillemin R., Vargo T., Rossier J., Minick S., Ling N., Rivier C., Vale W., Bloom F., Smith P. (1977). β-Endrophin and Adrenocorticotropin Are Secreted Concomitantly by the Pituitary Gland. Science.

[85] Parikh D., Hamid A., Friedman T.C., Nguyen K., Tseng A., Marquez P., Lutfy K. (2011). Stress-induced analgesia and endogenous opioid peptides: The importance of stress duration. Eur. J. Pharmacol..

[86] Apkarian A.V., Mutso A.A., Centeno M.V., Kan L., Wu M., Levinstein M., Banisadr G., Gobeske K.T., Miller R.J., Radulovic J. (2016). Role of adult hippocampal neurogenesis in persistent pain. Pain.

[87] Sauriyal D.S., Jaggi A.S., Singh N. (2011). Extending pharmacological spectrum of opioids beyond analgesia: Multifunctional aspects in different pathophysiological states. Neuropeptides.

[88] Bolijn S., Lucassen P.J. (2016). How the Body Talks to the Brain; Peripheral Mediators of Physical Activity-Induced Proliferation in the Adult Hippocampus. Brain Plast..

[89] Millan M.J., Przewlock R., Jerlicz M., Gramsch C., Höllt V., Herz A. (1981). Stress-induced release of brain and pituitary β-endorphin: Major role of endorphins in generation of hyperthermia, not analgesia. Brain Res..

[90] Xue L., Sun J., Zhu J., Ding Y., Chen S., Ding M., Pei H. (2020). The patterns of exercise-induced β-endorphin expression in the central nervous system of rats. Neuropeptides.

[91] Barfield E.T., Alexandra Moser V., Hand A., Grisel J.E. (2013). ß-Endorphin Modulates the Effect of Stress on Novelty-Suppressed Feeding. Front. Behav. Neurosci..

[92] Drake C.T., Chavkin C., Milner T.A. (2007). Opioid systems in the dentate gyrus. Prog. Brain Res..

[93] Padilla S.L., Reef D., Zeltser L.M. (2012). Defining POMC neurons using transgenic reagents: Impact of transient Pomc expression in diverse immature neuronal populations. Endocrinology.

[94] Bloom F., Battenberg E., Rossier J., Ling N., Guillemin R. (1978). Neurons containing β-endorphin in rat brain exist separately from those containing enkephalin: Immunocytochemical studies. Proc. Natl. Acad. Sci. USA.

[95] Wang D., He X., Zhao Z., Feng Q., Lin R., Sun Y., Ding T., Xu F., Luo M., Zhan C. (2015). Whole-brain mapping of the direct inputs and axonal projections of POMC and AgRP neurons. Front. Neuroanat..

[96] Porro C.A., Cavazzuti M., Baraldi P., Giuliani D., Panerai A.E., Corazza R. (1999). CNS pattern of metabolic activity during tonic pain: Evidence for modulation by β-endorphin. Eur. J. Neurosci..

[97] Wang J., Li X., Wu H., Ke J., Zhang Z., Wang Y. (2018). Effects of L-655,708 on expression changes of GABA, glutamate, and beta-endorphin induced by propofol anesthesia in rats. Eur. J. Inflamm..

[98] Houghten R.A., Swann R.W., Li C.H. (1980). beta-Endorphin: Stability, clearance behavior, and entry into the central nervous system after intravenous injection of the tritiated peptide in rats and rabbits. Proc. Natl. Acad. Sci. USA.

[99] Gao B., Hagenbuch B., Kullak-Ublick G.A., Benke D., Aguzzi A., Meier-Abt P.J. (2000). Organic anion-transporting polypeptides mediate transport of opioid peptides across blood-brain barrier. J. Pharmacol. Exp. Ther..

[100] Kumagai A.K., Eisenberg J.B., Pardridge W.M. (1987). Absorptive-mediated endocytosis of cationized albumin and a β-endorphin-cationized albumin chimeric peptide by isolated brain capillaries. Model system of blood-brain barrier transport. J. Biol. Chem..

[101] Rodríguez E.M., Blázquez J.L., Guerra M. (2010). The design of barriers in the hypothalamus allows the median eminence and the arcuate nucleus to enjoy private milieus: The former opens to the portal blood and the latter to the cerebrospinal fluid. Peptides.

[102] Hoffmann P., Terenius L., Thoren P. (1990). Cerebrospinal fluid immunoreactive fl-endorphin concentration is increased by voluntary exercise in the spontaneously hypertensive rat. Regul. Pept..

[103] Veening J.G., Gerrits P.O., Barendregt H.P. (2012). Volume transmission of beta-endorphin via the cerebrospinal fluid; a review. Fluids Barriers CNS.

[104] Leak R.K., Moore R.Y. (2012). Innervation of ventricular and periventricular brain compartments. Brain Res..

[105] Ableitner A., Schulz R. (1992). Neuroanatomical sites mediating the central actions of beta-endorphin as mapped by changes in glucose utilization: Involvement of mu opioid receptors. J. Pharmacol. Exp. Ther..

[106] Koehl M., Meerlo P., Gonzales D., Rontal A., Turek F.W., Abrous D.N. (2008). Exercise-induced promotion of hippocampal cell proliferation requires β-endorphin. FASEB J..

[107] Persson A.I., Naylor A.S., Jonsdottir I.H., Nyberg F., Eriksson P.S., Thorlin T. (2004). Differential regulation of hippocampal progenitor proliferation by opioid receptor antagonists in running and non-running spontaneously hypertensive rats. Eur. J. Neurosci..

[108] Nieto S.J., Quave C.B., Kosten T.A. (2018). Naltrexone alters alcohol self-administration behaviors and hypothalamic-pituitary-adrenal axis activity in a sex-dependent manner in rats. Pharmacol. Biochem. Behav..

[109] Persson A.I., Thorlin T., Bull C., Zarnegar P., Ekman R., Terenius L., Eriksson P.S. (2003). Mu- and delta-opioid receptor antagonists decrease proliferation and increase neurogenesis in cultures of rat adult hippocampal progenitors. Eur. J. Neurosci..

[110] Persson A.I., Thorlin T., Bull C., Eriksson P.S. (2003). Opioid-induced proliferation through the MAPK pathway in cultures of adult hippocampal progenitors. Mol. Cell. Neurosci..

[111] Zhang H., Torregrossa M.M., Jutkiewicz E.M., Shi Y.G., Rice K.C., Woods J.H., Watson S.J., Holden Ko M.C. (2006). Endogenous opioids upregulate brain-derived neurotrophic factor mRNA through δ- and μ-opioid receptors independent of antidepressant-like effects. Eur. J. Neurosci..

[112] de Oliveira M.S.R., da Silva Fernandes M.J., Scorza F.A., Persike D.S., Scorza C.A., da Ponte J.B., de Albuquerque M., Cavalheiro E.A., Arida R.M. (2010). Acute and chronic exercise modulates the expression of MOR opioid receptors in the hippocampal formation of rats. Brain Res. Bull..

[113] Neumaier J.F., Mailheau S., Chavkin C. (1988). Opioid receptor-mediated responses in the dentate gyrus and CA1 region of the rat hippocampus. J. Pharmacol. Exp. Ther..

[114] Drake C.T., Milner T.A. (1999). Mu opioid receptors are in somatodendritic and axonal compartments of GABAergic neurons in rat hippocampal formation. Brain Res..

[115] Svoboda K.R., Adams C.E., Lupica C.R. (1999). Opioid receptor subtype expression defines morphologically distinct classes of hippocampal interneurons. J. Neurosci..

[116] Tozuka Y., Fukuda S., Namba T., Seki T., Hisatsune T. (2005). GABAergic excitation promotes neuronal differentiation in adult hippocampal progenitor cells. Neuron.

[117] Schoenfeld T.J., Gould E. (2013). Differential Effects of Stress and Glucocorticoids on Adult Neurogenesis.

[118] Sahay A., Scobie K.N., Hill A.S., O’carroll C.M., Kheirbek M.A., Burghardt N.S., Fenton A.A., Dranovsky A., Hen R. (2011). Increasing adult hippocampal neurogenesis is sufficient to improve pattern separation HHS Public Access. Nature.

[119] Hill A.S., Sahay A., Hen R. (2015). Increasing Adult Hippocampal Neurogenesis is Sufficient to Reduce Anxiety and Depression-Like Behaviors. Neuropsychopharmacology.

[120] Culig L., Surget A., Bourdey M., Khemissi W., Le Guisquet A.M., Vogel E., Sahay A., Hen R., Belzung C. (2017). Increasing adult hippocampal neurogenesis in mice after exposure to unpredictable chronic mild stress may counteract some of the effects of stress. Neuropharmacology.

[121] Izquierdo I., Souza D.O., Carrasco M.A., Dias R.D., Perry M.L., Eisinger S., Elisabetsky E., Vendite D.A. (1980). Beta-endorphin causes retrograde amnesia and is released from the rat brain by various forms of training and stimulation. Psychopharmacology.

[122] Heybach J.P., Vernikos J. (1981). Naloxone inhibits and morphine potentiates the adrenal steroidogenic response to ACTH. Eur. J. Pharmacol..

[123] Miller B.R., Hen R. (2015). The current state of the neurogenic theory of depression and anxiety. Curr. Opin. Neurobiol..

[124] Eisch A.J., Barrot M., Schad C.A., Self D.W., Nestler E.J. (2000). Opiates inhibit neurogenesis in the adult rat hippocampus. Proc. Natl. Acad. Sci. USA.

[125] Molina V.A., Heyser C.J., Spear L.P. (1994). Chronic variable stress or chronic morphine facilitates immobility in a forced swim test: Reversal by naloxone. Psychopharmacology.

[126] degli Uberti E.C., Petraglia F., Bondanelli M., Guo A.L., Valentini A., Salvadori S., Criscuolo M., Nappi R.E., Genazzani A.R. (1995). Involvement of μ-opioid receptors in the modulation of pituitary-adrenal axis in normal and stressed rats. J. Endocrinol. Investig..

[127] Ra S.M., Kim H., Jang M.H., Shin M.C., Lee T.H., Lim B.V., Kim C.J., Kim E.H., Kim K.M., Kim S.S. (2002). Treadmill running and swimming increase cell proliferation in the hippocampal dentate gyrus of rats. Neurosci. Lett..

[128] Hayward M.D., Pintar J.E., Low M.J. (2002). Selective reward deficit in mice lacking β-endorphin and enkephalin. J. Neurosci..

[129] Karlsson R.M., Wang A.S., Sonti A.N., Cameron H.A. (2018). Adult neurogenesis affects motivation to obtain weak, but not strong, reward in operant tasks. Hippocampus.

[130] McGonigle C.E., Nentwig T.B., Wilson D.E., Rhinehart E.M., Grisel J.E. (2016). Β-Endorphin Regulates Alcohol Consumption Induced By Exercise Restriction in Female Mice. Alcohol.

[131] He Y., Lu Y., Shen Y., Wu F., Xu X., Kong E., Huang Z., Sun Y., Yu W. (2019). Transgenic increase in the β-endorphin concentration in cerebrospinal fluid alleviates morphine-primed relapse behavior through the μ opioid receptor in rats. J. Med. Virol..

[132] Goldfarb A.H., Jamurtas A.Z., Kamimori G.H., Hegde S., Otterstetter R., Brown D.A. (1998). Gender effect on beta-endorphin response to exercise. Med. Sci. Sports Exerc..

[133] Kanarek R.B., Gerstein A.V., Wildman R.P., Mathes W.F., D’Anci K.E. (1998). Chronic running-wheel activity decreases sensitivity to morphine-induced analgesia in male and female rats. Pharmacol. Biochem. Behav..

[134] Hare B.D., Beierle J.A., Toufexis D.J., Hammack S.E., Falls W.A. (2014). Exercise-associated changes in the corticosterone response to acute restraint stress: Evidence for increased adrenal sensitivity and reduced corticosterone response duration. Neuropsychopharmacology.

[135] White-Welkley J.E., Bunnell B.N., Mougey E.H., Meyerhoff J.L., Dishman R.K. (1995). Treadmill exercise training and estradiol differentially modulate hypothalamic-pituitary-adrenal cortical responses to acute running and immobilization. Physiol. Behav..

[136] Rahimi S., Peeri M., Azarbayjani M.A., Anoosheh L., Ghasemzadeh E., Khalifeh N., Noroozi-Mahyari S., Deravi S., Saffari-Anaraki S., Hemat Zangeneh F. (2020). Long-term exercise from adolescence to adulthood reduces anxiety- and depression-like behaviors following maternal immune activation in offspring. Physiol. Behav..

[137] Duman C.H., Schlesinger L., Russell D.S., Duman R.S. (2008). Voluntary exercise produces antidepressant and anxiolytic behavioral effects in mice. Brain Res..

[138] Barha C.K., Falck R.S., Davis J.C., Nagamatsu L.S., Liu-Ambrose T. (2017). Sex differences in aerobic exercise efficacy to improve cognition: A systematic review and meta-analysis of studies in older rodents. Front. Neuroendocrinol..

[139] Ma X., Hamadeh M.J., Christie B.R., Foster J.A., Tarnopolsky M.A. (2012). Impact of treadmill running and sex on hippocampal neurogenesis in the mouse model of amyotrophic lateral sclerosis. PLoS ONE.

[140] Cahill S.P., Cole J.D., Yu R.Q., Clemans-Gibbon J., Snyder J.S. (2018). Differential Effects of Extended Exercise and Memantine Treatment on Adult Neurogenesis in Male and Female Rats. Neuroscience.

[141] Pluchino N., Drakopoulos P., Casarosa E., Freschi L., Petignat P., Yaron M., Genazzani A.R. (2015). Effect of estetrol on beta-endorphin level in female rats. Steroids.

[142] Bernardi F., Pluchino N., Pieri M., Begliuomini S., Lenzi E., Puccetti S., Casarosa E., Luisi S., Genazzani A.R. (2006). Progesterone and medroxyprogesterone acetate effects on central and peripheral allopregnanolone and beta-endorphin levels. Neuroendocrinology.

[143] Meyer W.R., Muoio D., Hackney T.C. (1999). Effect of sex steroids on β-endorphin levels at rest and during submaximal treadmill exercise in anovulatory and ovulatory runners. Fertil. Steril..

[144] Schneider A.M., Simson P.E., Spiller K., Adelstein J., Vacharat A., Short K.R., Kirby L.G. (2009). Stress-dependent enhancement and impairment of retention by naloxone: Evidence for an endogenous opioid-based modulatory system protective of memory. Behav. Brain Res..

[145] Holmes M.M., Galea L.A.M. (2002). Defensive behavior and hippocampal cell proliferation: Differential modulation by naltrexone during stress. Behav. Neurosci..

[146] Brito R.G., Rasmussen L.A., Sluka K.A. (2017). Regular physical activity prevents development of chronic muscle pain through modulation of supraspinal opioid and serotonergic mechanisms. Pain Rep..

[147] Glasper E.R., Schoenfeld T.J., Gould E. (2012). Adult neurogenesis: Optimizing hippocampal function to suit the environment. Behav. Brain Res..

[148] Cameron H.A., Schoenfeld T.J. (2018). Behavioral and structural adaptations to stress. Front. Neuroendocrinol..

